# Long-term follow-up of beryllium sensitized workers from a single employer

**DOI:** 10.1186/1471-2458-10-5

**Published:** 2010-01-04

**Authors:** Mona Duggal, David C Deubner, Anne M Curtis, Mark R Cullen

**Affiliations:** 1 Department of Internal Medicine, Yale University School of Medicine, New Haven, CT, USA; 2 Brush-Wellman Inc., 14710 West Portage River South Road, Elmore OH, 43416, USA; 3 Department of Diagnostic Imaging, Yale University School of Medicine, New Haven, CT, USA; 4 Stanford University, Department of General Internal Medicine, 251 Campus Drive, MSOB 338 Stanford, CA, 94306, USA

## Abstract

**Background:**

Up to 12% of beryllium-exposed American workers would test positive on beryllium lymphocyte proliferation test (BeLPT) screening, but the implications of sensitization remain uncertain.

**Methods:**

Seventy two current and former employees of a beryllium manufacturer, including 22 with pathologic changes of chronic beryllium disease (CBD), and 50 without, with a confirmed positive test were followed-up for 7.4 +/-3.1 years.

**Results:**

Beyond predicted effects of aging, flow rates and lung volumes changed little from baseline, while D_L_CO dropped 17.4% of predicted on average. Despite this group decline, only 8 subjects (11.1%) demonstrated physiologic or radiologic abnormalities typical of CBD. Other than baseline status, no clinical or laboratory feature distinguished those who clinically manifested CBD at follow-up from those who did not.

**Conclusions:**

The clinical outlook remains favorable for beryllium-sensitized individuals over the first 5-12 years. However, declines in D_L_CO may presage further and more serious clinical manifestations in the future. These conclusions are tempered by the possibility of selection bias and other study limitations.

## Background

The beryllium lymphocyte proliferation test (BeLPT), originally developed as a diagnostic tool for chronic beryllium disease (CBD) [1,2], came into widespread use in the 1990s as a surveillance test among asymptomatic, clinically healthy, beryllium exposed workers [3-8]. Since empiric evidence for screening efficacy has been lacking, the choice was based on cross-sectional observations [4-6] and anecdotal reports of progression to CBD in BeLPT-positive workers initially free of clinical manifestations [9]. It has been the working presumption that some, or all of those sensitized to beryllium would develop CBD in the future [3,10-12]. Rates of confirmed positive tests varied among populations tested, ranging from 2-12% [3,4,6-8,13,14]. Given the recent estimate of as many as 134,000 workers currently exposed to beryllium in the U.S. [15], and the likelihood that many more were exposed in the past, these data and inferences together would suggest a large potential burden of CBD if more than a small percentage developed clinical manifestations. Since the disease as classically described is highly morbid and carries a high mortality rate [16,17], screening has generated a challenging set of issues confronting clinicians who may care for identified "positives," as well as questions regarding the efficacy of screening.

Despite wide application of the test in industry and research studies, however, longitudinal observations to document and quantify the natural history after initial sensitization, or to identify factors that might confer better or worse clinical trajectories, remain scant. Only one such study has been published to date [18]. In this, Newman and colleagues reported results of clinical and pathologic re-evaluation on a series of 55 BeLPT-positive volunteers initially free of granulomas or comparable inflammatory changes in the lung based on BAL and biopsy. Almost one-third subsequently developed evidence of lung pathology compatible with CBD, after an average follow-up of 4.8 years. The authors interpreted this as evidence supporting the presumption that a high fraction of BeLPT-positive subjects will eventually develop CBD, although their clinical data demonstrated that few of their patients had clinical findings typical of CBD, and only one was on treatment with systemic corticosteroids [19].

The present study was designed to contribute further knowledge about long term outcome after a confirmed positive BeLPT test during routine surveillance of exposed workers, including both those with and without evidence of CBD on bronchoscopy and/or bronchoalveolar lavage at the time of initial surveillance. We offered follow-up X-rays, lung function tests, physical exams, questionnaires and repeat BeLPT testing to all former and present employees of a single large beryllium company identified as being BeLPT-positive during workplace surveys conducted between 1992 and 2001. Based on the results we have documented physiologic course and estimated the rate of clinically apparent CBD at follow-up, and determined risk factors, if any, for that adverse outcome.

## Methods

### Study Population

The cohort comprised both former and current workers at plants in Elmore, Ohio, Reading, Pennsylvania and Tucson, Arizona who were positive on the BeLPT surveillance test between 1992 and 2001 during employment, and had at least one confirmatory test. The parent company provided contact information for 185 employees initially identified. Three respondents were subsequently deemed ineligible because of diagnoses made clinically before surveillance testing and excluded. The resulting group yielded the same population described by Donovan et al except for the inclusion in our recruitment group of 5 additional BeLPT positive and confirmed subjects identified at Tucson after 2000, and the exclusion of the Delta plant positives included in their paper but whose numbers we considered too few to follow-up for practical reasons[20]. An invitation to participate was mailed to the last known address. Searches were made for subjects whose initial letter was returned as undeliverable.

Plant medical records of consented subjects were abstracted, as were records of the independent examinations for CBD that had been recommended for all confirmed positives and completed by most at the time of initial screening. That "baseline" exam included bronchoscopy with bronchoalveolar lavage (differential cell counts and BeLPT), and biopsy. Baseline lung function values were inferred from the studies obtained at the specialty exam. In addition, summary demographic data and baseline lung function were provided on non-participants to address selection bias.

Subjects who agreed to participate were invited for new "follow-up" examinations. Clinics near plant sites were contracted to perform a standardized physical examination, obtain a postero-anterior and lateral chest X-ray, administer a questionnaire, draw and send blood to two labs for BeLPT, and conduct pulmonary spirometry, total lung capacity and diffusion studies.

The protocol was approved by the Human Investigation Committee of the Yale University School of Medicine.

### BeLPT test

A blood sample was obtained and split, with one each subsequently sent to National Jewish Medical and Research Center (NJMRC; Denver, CO) and Specialty Laboratories, (La Jolla, CA). Assays were performed as previously published [3](Kreiss et al.1989).

### Pulmonary Physiologic Assessment

Clinics differed in their equipment, but each followed ATS quality guidelines [21,22]. Knudson predicted norms were used for lung volumes and flow rates [23]. The predicted values of Crapo and Morris were used for D_L_CO assessment [24]. To investigate possible effects of inter-laboratory differences, serial lung function studies obtained at the Ohio plant medical department were abstracted on the subset of workers active at the plant between 1994 and 2001 when D_L_CO testing was discontinued on a routine basis.

### Chest Radiographs

An experienced chest radiologist (A.M.C., a NIOSH-certified B reader) interpreted all chest radiographs--including baseline, follow-up and interval films where available--side-by-side, unblinded to film date. A chest x-ray consistent with CBD was defined as having small rounded opacities of profusion grade ≥1/0 based on the ILO classification system, and/or mediastinal or hilar lymphadenopathy [17,25].

### Health and Exposure Survey

A specially developed questionnaire assessed post-screening employment, medical history, history of atopy and smoking status and incorporated a modified version of the standardized American Thoracic Society respiratory questionnaire for cough, phlegm, wheeze, and breathlessness. Exposure to beryllium was assessed by years worked, time since leaving the exposure, and whether or not they remained employed in a workplace using beryllium after initially screening positive.

### Classification of Baseline Status

Subjects had been classified as **beryllium sensitized **(BeS) if they had two or more abnormal BeLPT tests and no evidence of granulomas and/or mononuclear cell infiltrates on trans-bronchial lung biopsy at the time of baseline evaluation [17,25]. Subjects were classified as having surveillance detected **CBD **if they had, in addition, granulomas and/or mononuclear cell infiltrates in lung tissue, or had lymphocytosis (>20%) and positive BeLPT on BAL. The small number of sensitized workers who initially refused bronchoscopy but had no clinical abnormalities were also classified as **BeS**.

### Criteria for Clinical Abnormality at Follow-up

We defined a subject as having a clinical abnormality consistent with CBD if the manifested one or more of the following changes on follow-up exam: 1) Chest X-ray changes typical of CBD; 2) Total lung capacity and/or FVC <80% predicted; 3) D_L_CO <80% predicted absent COPD, or 4) Treatment with oral steroids for CBD by a physician.

Note: Subjects were considered to have COPD if: 1) they were former or current smokers, *and *2) met GOLD criteria for Stage II (FEV_1_/FVC less than 70% and FEV_1 _< 80% predicted), *and *3) had evidence of air-trapping or bullous disease on chest X-ray and/or total lung capacity >100% predicted.

### Statistical Analysis

Chi-square and Student t-tests were used to compare the recruited subjects with those who did not respond or elected not to participate. Linear regression techniques and analysis of variance were used to assess lung function changes. Development of clinically manifest CBD was estimated for the entire group, and separately for BeS and CBD subgroups, using the Kaplan-Meier survivor functions. Candidate predictors for clinical signs included: duration of follow-up, demographics, work history, atopy, smoking history, baseline functional status and follow-up BeLPT results (i.e., persistent beryllium sensitivity). Continuous variables were compared using Student's t-test. Categorical characteristics were compared between subgroups using Fisher's exact test. Differences for all comparisons were considered significant when p < 0.05 in a 2-sided test. Analysis was performed using Stata version 8.0 (Stata Corporation, College Station, TX, USA).

## Results

### Recruitment and Baseline Characteristics

**Figure 1:**
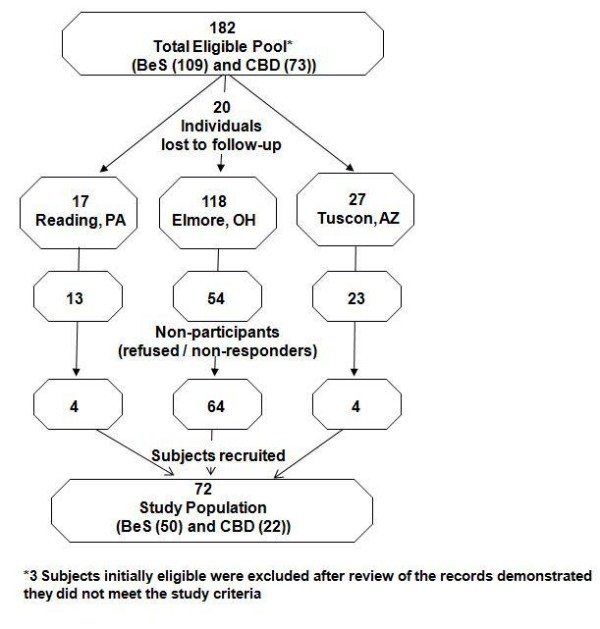
**Cohort Recruitment Schema**.

Of the 182 eligible subjects with confirmed positive BeLPT tests at one of the waves of workplace screening between 1992 and 2001, 72 (40%) agreed to participate overall. This represented a 54% response at the largest (Ohio) facility, but <25% at the two smaller sites. Subjects with CBD diagnosed at baseline were significantly less likely to participate, p < .02. Recruitment is depicted in Figure 1: Cohort recruitment schema.

Table 1 shows the baseline characteristics of the participants at the time of their initial identification as BeLPT positive. Compared to non-participants, the proportion of females and still-active at the plant was the same, but whites were more likely than others to participate, p < .01. Mean age among those who participated was higher (4.4 years on average), but adjusted for age, baseline lung function was almost identical on every parameter including D_L_CO (data not shown).

**Table 1:** Baseline demographic and exposure profile of participants initially classified as Chronic Beryllium Disease (CBD) and Beryllium Sensitization (BeS), with and without those with COPD

	BeS COPD in (n = 50)	CBD COPD in (n = 22)	P value	BeS COPD out (n = 40)	CBD COPD out (n = 20)	P value
Age in years (s.d.)	44.2 (11.1)	47.8 (10.5)	0.20	42.6 (11.2)	46.7 (10.2)	0.17

Sex (%male)	78	77	0.95	73	80	0.53

Race (% white)	94	100	0.71	93	100	0.66

Smoking Status						
Non-smoker%	42	36		50	40	

Ex smoker %	20	36	0.32	18	40	0.16

Current smoker %	38	27		33	20	

Mean length from hire date to baseline evaluation in years (s.d)	12.4 (11.1)	13.7 (7.0)	0.60	11.6 (10.1)	14.3 (7.0)	0.28

Period of beryllium exposure in years (s.d)	15.6 (11.8)	18.0 (8.4)	0.41	15.1 (11.7)	18.7 (8.5)	0.22

Time from baseline to follow-up evaluation(s.d)	7.1 (3.0)	8.1 (3.4)	0.20	7.0 (3.0)	8.5 (3.4)	0.09

History of hay fever, asthma or eczema (% positive)	48	50	0.96	50	45	0.72

**Pulmonary Function Tests**						

FEV_1 _mean % predicted (s.d.)	89.3 (14.5)	86.2 (16.7)	0.43	92.6 (12.7)	89.1 (14.3)	0.33

FVC mean % predicted (s.d.)	96.1 (14.8)	88.8 (11.7)	0.04	97.9 (14.3)	89.6 (11.5)	0.03

FEV_1_/FVC mean % predicted (s.d)	92.8 (10.3)	95.0 (12.0)	0.44	95.6 (8.1)	97.0 (8.1)	0.52

D_L_CO mean % predicted (s.d.)	101.3 (16.1)	99.9 (23)	0.77	103.6 (14.2)	104.0 (20.1)	0.94

TLC mean %(predicted) (s.d.)	98.6 (14.6)	94.5 (14.5)	0.35	98.6 (14.3)	91.5 (12.0)	0.10

Clinical evidence of COPD N (%)	10 (20)	2 (9.1)	0.32			

* 3 subjects initially eligible were excluded after record review as they did not meet study criteria

Subjects were further stratified based on the outcome of their initial complete evaluation as "Beryllium Sensitized" (BeS), implying no pathologic evidence of CBD was found on biopsy and BAL (N = 44) or the test was not performed at baseline (N = 6); or CBD (N = 22). As can be seen, the groups did not differ appreciably on demographic characteristics, length of work experience, smoking, allergy history or lung function, although those with CBD had lower FVC. Not demonstrated, ever-smokers in each subgroup had significantly lower FEV_1 _and FEV_1_/FVC levels than non-smokers; current smokers had lower D_L_COs. Twelve smokers had moderate or severe COPD, 10 (20%) of the BeS subjects and 2 (9%) with CBD, a non-significant difference in rate, p = 0.32. Table 1 shows the baseline characteristics of the two groups with and without these subjects included.

### Clinical Outcome at Follow-Up

Table 2 shows the changes in lung function from baseline examination to follow-up. In 8 subjects the total lung capacity and/or D_L_CO could not be calculated because of patient cooperation or technical problems.

**Table 2:** Mean percent predicted values for physiologic parameters at baseline and at follow-up for each clinical subgroup (p < .05 for differe ce in *italics*)

Pulmonary Function Tests		BeS COPD In	BeS COPD out	BeS COPD	CBD COPD In	CBD COPD out	CBD COPD
N		50	40	10	22	20	2

FEV_1 _%	Before	89.3	92.6	76.2	86.2	89.1	57.9

	After	88.2	91.1	77.0	82.7	86.2	48.4

	**Difference**	**1.1**	**1.6**	**-0.8**	**3.5**	**2.9**	**9.5**

FVC %	Before	96.1	97.9	89.1	88.8	89.6	80.9

	After	94.4	94.6	93.5	87.0	88.9	67.5

	**Difference**	**1.8**	**3.3**	**-4.4**	**1.9**	**0.7**	**13.5**

FEV_1_/FVC %	Before	92.8	95.6	81.8	95.0	97.0	74.5

	After	92.9	95.6	82.0	95.0	98.0	64.9

	**Difference**	**-0.0**	**-0.0**	**-0.2**	**-0.0**	**-1.0**	***9.6***

D_L_CO mean %*	Before	101.3	103.6	92.2	99.9	104.0	59.4

	After	85.7	87.8	75.5	79.6	83.4	41.8

	**Difference**	***16.0***	***15.8***	***16.7***	***20.3***	***20.6***	***17.6***

TLC mean %*	Before	98.6	98.6	98.7	94.5	91.5	125.1

	After	94.5	93.5	98.2	86.4	85.6	94.3

	**Difference**	**4.2**	**5.1**	**0.5**	**8.1**	**5.9**	***30.8***

* Data missing for 8 subjects

**Figure 2:**
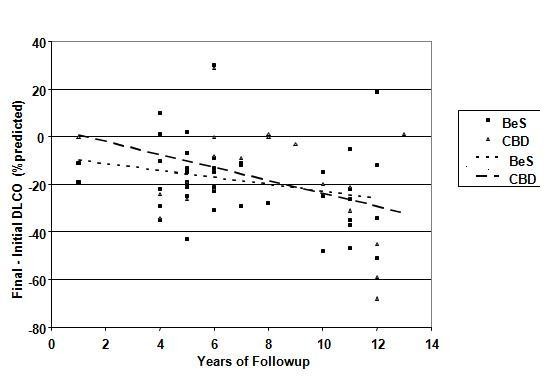
**Difference in DLCO percent predicted (Crapo) between baseline and follow-up visits versus time between examinations**. Linear regression lines are depicted for the entire group and each of the subgroups based on baseline lung evaluation.

Over an average of 7.4 +/- 3.1 years there was little change in the mean % of predicted for FEV_1 _(-1.8%), FVC (- 1.8%), or FEV_1_/FVC (+ 0.04%), although ever-smokers lost function on each parameter significantly faster than non-smokers (data not shown). The change in TLC % predicted was larger, but similar across the groups except for the two persons with CBD and COPD. In contrast, the final-initial difference in D_L_CO % predicted was significant and large: - 17.3%. This decline was similar across all groups, BeS and CBD, with and without COPD. Analysis of variance showed that declines were equivalent at all three sites. There was no significant relationship between change in D_L_CO and baseline status, BeLPT at follow-up, duration of beryllium exposure or smoking, but these was a significant positive correlation (p < .02) between loss and length of follow-up as illustrated in Figure 2.

BeS subjects lost at a rate of 1.4% per year, while those with lung involvement at baseline lost an average of 2.7% annually, but this difference was not significant. Neither percent lymphocytes on initial BAL, nor biopsy findings, added greater predictive value regarding D_L_CO decline. Notably, the 19 subjects followed continuously at the plant lung function laboratory between 1994 and 2001 lost a mean of 9.3% percent predicted during this time (+/- SD 14.5%, range 44% loss to 15% gain).

Because of concern about drawing strong inferences based on D_L_CO changes alone, since pre- and post- values were obtained in different laboratories for all subjects and compared only at two points in time, we evaluated the cases also from a clinical perspective, classifying each separately using the above stated criteria. This seemed as well another way of managing the possible confounding effects of COPD, which was prevalent among study subjects as noted. By our criteria, 8 (11.1%) of the 72 subjects demonstrated one or more clinical manifestations of CBD based on typical X-ray changes (N = 2), a pattern of restrictive/interstitial lung dysfunction (N = 4), abnormal D_L_CO (N = 6), or some combination thereof. In only two was a low D_L_CO the sole criterion for inclusion, so this result is, in part at least, independent of the observation regarding D_L_CO loss. Results of follow-up examinations for these 8 individuals are summarized in Table 3. As shown, patient 7 had no clear clinical manifestation of CBD, but had been started on oral steroids for presumed CBD by his physician; he was unable to complete the TLC and D_L_CO testing at follow-up.

**Table 3:** Descriptive characteristics and clinical evaluation results of participants with clinical Chronic Beryllium Disease at follow-up

Parameter	Pt.1	Pt.2	Pt. 3	Pt.4	Pt 5	Pt 6	Pt.7	Pt.8
**Baseline diagnosis**	CBD	CBD	CBD	CBD	CBD	CBD	CBD	BeS

Baseline to current evaluation, in yrs.	12	5	12	4	1	9	11	7

Yrs. since first worked at site to initial evaluation	4	10	23	1	26	22	8	18

Yrs. of beryllium exposure	15	12	29	2	27	36	10	25

Yrs. since last worked at site	6	3	6	3	0	9	10	0

Smoking Status	Never smoker	Never smoker	Ex smoker	Ex smoker	Ex smoker	Ex smoker	Ex smoker	Current smoker

No. of pack years	0	0	9	20	11	71	8	21.5

Steroid Use	No	Yes (Inhalational)	No	Yes (Inhalational)	No	Yes (Inhalational)	Yes (oral)	No

BeLPT result (Lab 1/Lab 2)	+/+	+/-	+/-	+/+	+/+	-/-	+/-	+/+

**Pulmonary Function tests (% predicted)**								

FEV_1_								

Initial	73	87	81	85	65	95	72	87

Final	62	93	79	89	63	85	70	71

FVC								

Initial	80	78	82	97	71	121	83	89

Final	62	87	82	96	69	89	79	68

FEV_1_/FVC								

Initial	92	111	100	88	92	78	87	97

Final	99	107	98	92	89	94	88	106

TLC								

Initial	88	78	97	107	95	96	95	88

Final	80	58	88	91	87	89	N/A	79

D_L_CO								

Initial	108	95	124	98	N/A	97	131	116

Final	49	69	56	64	53	62	N/A	82

**X-ray Changes or CBD**	Bilateral hilar adenopathy and diffuse small opacities	None	None	None	None	Borderline enlargement of lymph nodes	None	None

**Basis for Clinical CBD**	X-ray, Low D_L_CO, Low FVC	Low TLC, Low D_L_CO	Low D_L_CO	Low D_L_CO	Low FVC, Low D_L_CO	X-ray, Low D_L_CO	On oral steroids for presumed clinical CBD	Low FVC, Low TLC

Table 4 depicts the follow-up data for the entire group, stratified by baseline category. As can be appreciated, there were differences in the fate of the two groups. About 3/4 of those with CBD at baseline remained positive on the BeLPT in one or both labs, compared to 42% of the those with BeS at baseline (p = 0.035). More strikingly, while 7 of 22 with CBD demonstrated one or more clinical manifestations of CBD (31.8%) at follow-up, only 1 of the 50 (2%) with BeS at baseline met the criteria, a significant difference, p < .001. Using Kaplan-Meier survivor analysis of the two groups from the time of initial positive screening to the time of follow-up, this difference was also highly significant, p < .002.

**Table 4:** Follow up Clinical Examination Results

Questionnaire variables	BeS COPD in (n = 50)	CBD COPD in (n = 22)	P value	BeS COPD out (n = 40)	CBD COPD out (n = 20)	P value
History of hay fever, asthma or eczyma (% positive)	48	50	0.96	50	45	0.72

Steroid use (Inhaled or Oral) %	4	32	0.001	5	25	0.023

Smoking Status						

Nonsmoker %	42	36		48	40	

Ex smoker %	42	55	0.56	38	55	0.32

Current smoker %	16	9		15	5	

% still working at beryllium-using facility	36	23	0.27	40	20	0.12

Current BeLPT test results %*						

(+/+)	17	43	0.025	21	42	0.03

(+/-)	26	33		21	37	

(-/-)	57	24		58	21	

Clinical CBD N (%)	1(2.0%)	7(31.8%)	0.001	1(2.5)	7(35.0)	0

**Pulmonary Function Tests (% predicted)**						

FEV_1 _(s.d)	88.2(17.4)	82.7(21.3)	0.25	91.1(13.0)	86.2(16.7)	0.22

FVC (s.d.)	94.4 (18.5)	87.0(18.4)	0.12	94.6(14.0)	88.9 (15.7)	0.14

FEV_1_/FVC (s.d)	92.9 (10.2)	95.0 (14.1)	0.47	95.6(8.4)	98.0(9.6)	0.32

D_L_CO (s.d.)	85.4(20.8)	79.6(20.8)	0.30	87.8(15.9)	83.4(17.4)	0.36

TLC (s.d.)	94.5(19.1)	86.4(14.4)	0.05	93.5(14.6)	85.6(14.7)	0.07

*9 participants had either only 1 or no BeLPT test(4 had 1 test and 5 had no test result)

### Predictors of Clinical Disease at Follow-up

The strongest predictor of clinical manifestations of CBD at follow-up was the presence of CBD related pathology in the lung at the time of initial evaluation. Specific biopsy changes made no difference, nor did the percent lymphocytes or granulocytes on initial BAL. Among those with initial changes, 31.8% had clinical abnormalities typical of CBD at follow-up, raising the question whether any characteristic(s) determined at baseline, or subsequently, might be associated with risk. Demographics, baseline clinical status, allergy history, smoking history, prior beryllium exposure (measured crudely as years worked in the plant), continuation of work around beryllium, results of the BAL, and persistent blood BeLPT reactivity were all considered. As can be seen in Table 5, none appear strongly associated, although the clinical CBD cases did have, on average, lower FVC and FEV_1 _at the outset than those who did not manifest such changes.

**Table 5:** Predictors of clinical disease among participants with CBD at baseline

Baseline Characteristics	WITHOUT clinical CBD at follow-up n = 15	WITH clinical CBD at follow-up n = 7*	P value
Age in years (s.d.)	45.6 (9.27)	52.6 (12.0)	0.15

Race (% white)	93	100	0.48

Sex (%male)	73	86	0.52

History of hay fever, asthma or eczema %	47	57	0.65

Mean length from hire date to baseline evaluation, in years (s.d)	13.7 (5.9)	13.9 (9.4)	0.95

Period of beryllium exposure, in years (s.d)	18.6 (8.1)	16.6 (9.7)	0.61

Time from baseline to follow-up evaluation(s.d)	8.3 (2.9)	7.7 (4.4)	0.70

**Baseline PFTs (% predicted (s.d.))**			

FEV_1_	87.5 (16.3)	83.4 (18.3)	0.60

FVC	91.2 (12.2)	83.7 (9.3)	0.17

FEV_1_/FVC	95.9 (13.1)	92.9 (9.9)	0.60

D_L_CO	98.1 (23.9)	104.3 (22.0)	0.59

TLC	95.8 (17.2)	92.4 (9.0)	0.64

**Follow-up Characteristics**			

**Smoking Status**			

Nonsmoker %	40	29	

Ex smoker %	47	71	0.44

Current smoker %	13	0	

% still working at beryllium facility	27	14	0.52

BeLPT results *			

(+/+)	40	50	

(+/-)	33	33	0.89

(-/-)	27	17	

* result for 1 person was missing			

D_L_CO levels, which proved most often abnormal at follow-up, were almost identical between the groups at the outset. By way of caution, however, neither quantitative beryllium exposure, nor HLA genotype, each considered *a priori *relevant as predictors of long-term outcome, was available for this comparison. Moreover, the number of CBD cases was too small to exclude some beneficial effects, for example, from leaving beryllium exposure or quitting smoking.

### BeLPT Results at Follow-Up

Sixty-three of the subjects had repeat BeLPT testing on a split sample at two separate labs--National Jewish and Specialty Lab. Five subjects refused to be tested, and in 4 others, a satisfactory test was unable to be performed by one lab or the other. Only 15, (24.1%) were positive at both labs on follow-up while 19 were positive in one lab but negative in the other. Thirty-three subjects were negative. Overall agreement between the labs, as shown on Table 6, was almost 68%, with a Kappa of 0.43, significantly different from "random," p < .0008.

**Table 6:** Comparison at follow-up of split sample blood for BeLPT

	Lab 1	
Lab 2	+ ve	-ve
**+ ve**	15	12
**- ve**	7	29
**K = 0.43, Agreement = 68.06%, P = 0.0008**		

## Discussion

To our knowledge this is the first reported clinical follow-up of a population-based groups of subjects identified as being blood BeLPT positive on routine workplace surveillance. Overall, the findings are mixed. On the one hand, this study shows that the fraction of subjects who have developed objective clinical manifestations typical of CBD, at 11.1%, is low. Moreover, from a clinical perspective the severity in each of the 8 cases observed is mild to moderate; only one of the patients has been treated with systemic corticosteroids by their physician, although each is under the care of a pulmonologist. For the subset of subjects who proved negative on initial full lung evaluation, the results appear even more favorable; only 1 of 50 meets our criteria for clinical CBD. Half of these subjects, unlike most who had CBD at the outset, have lost their BeLPT positivity.

On the other hand, while few subjects met criteria for CBD, there appears to be a drop in % predicted D_L_CO in many more subjects, including those without established lung involvement at baseline. Although not associated with group changes in lung volumes or flow rates, three lines of evidence suggest these decrements are not merely an artifact of the fact that pre and post testing was conducted in different labs, nor that only two data points were used to calculate change. First, the average drop of 17.4% was almost identical for subjects at each testing site, which speaks against lab variation as the major explanation. Second, the significant trend with time since initial BeLPT positivity (Figure 2)--but not age or duration of exposure--would seem most consistent with progressive, albeit slow loss. As noted, those with CBD at baseline appear to be losing faster than those without, on average. Third, among the subset of still active workers between 1994 and 2001 continuously tested at the plant, losses averaged well over 1% decline from predicted per year.

Unfortunately, we were unable to delineate any factor or factors other than baseline pathology that predicted rate of loss or development of clinical signs. However, two of the most important potential factors, smoking cessation and cessation of all beryllium exposure, would only have been noticeable with an overwhelming effect since the population size is too small. Two other factors considered relevant, extent of prior exposure to beryllium and HLA-DP polymorphism [10,11], could not yet be evaluated because we have not linked our results with sources for these data.

Beyond these, there are some other important limitations of our study. Most obviously, although we recruited 54% of the eligible cohort members at the largest site, recruitment overall was less than hoped (72/182 identified) so we cannot be certain that no selection bias was introduced. In theory, subjects with better or worse outcomes could have selectively chosen to either participate or not, although except for the lesser participation of younger workers and non-whites, baseline characteristics were very similar between those who did and did not come. Notwithstanding our efforts to communicate the independence of the investigators from the employer-sponsor, all subjects were aware of our funding source. This may have influenced some subjects' decisions introducing bias in either direction in theory,

Another important limitation of this study was the reliance on clinical observations to establish key end-points of the study, namely the paired chest X-rays and paired lung function tests--done in different labs. Most problematic were the lung function studies. While all attempts were made to follow ATS procedures [21,22] at follow-up exams, we were unable to verify the procedures where baseline respiratory examinations were conducted, though most were in the laboratories of university medical centers. Furthermore, the high prevalence of COPD in the study population by our definition (N = 12, 16.7%), whose changes over the follow-up period could potentially confound group interpretation of the physiologic data, dictated that we use a clinical end-point for the study rather than rely strictly on group physiologic observations. In point of fact, however, each of the cases were, on review, easily classifiable despite the fact that diminished D_L_CO was the most prevalent reason for classification as CBD (present in 6 subjects and the only basis for classification in 2, see Table 3). Still, some physiologic contribution by CBD in the 12 patients with COPD cannot be ruled out. This possibility underscores a final limitation, namely that we did not, like Newman [18](Newman et al, 2005), repeat the biologic assessment for pathologic or BAL changes of CBD; some of those initially classified as BeS may have evolved over time, and subsequently be, theoretically, at greater risk for clinical disease.

Where does this new data leave us in the effort to control CBD? First, we have affirmed our earlier impression that the BeLPT, despite its limitations [26,27] (Table 5), detects subjects at some risk for clinical disease once confirmed by a second positive test. In fact, some of those detected at screening already had evidence of clinically manifest disease at first recognition (cf patients 2 and 5, Table 3), suggesting that the often used term "sub-clinical CBD" may be less precise for describing such patients than an alternative designation such as "surveillance-detected CBD." Whether BeLPT positivity (confirmed) carries similar implications among individuals whose exposures may have been more limited is uncertain, as our subjects were all workers in primary beryllium-related occupations. Also uncertain is the benefit of identifying at-risk individuals in the absence of any action known to alter their clinical outcome.

When testing is performed, the optimal approach to follow-up of confirmed BeLPT positives also remains unresolved. Full pulmonary evaluation, with bronchoscopy, biopsy, and BAL, may be justified given the suggestion of a different prognosis for those with vs. those without lung involvement, but this benefit remains unproved. Although we continue to support removal from continued occupational beryllium exposure based on a precautionary point of view, we would be hard pressed to take a more rigid position based on our results in a case where an individual chose to remain in exposed work, as many of our subjects in this study did, despite advice and a generous economic package, without obviously greater risk than those who chose to leave. Hopefully, given the continued interest in CBD, better scientific foundations for these clinical and public health decisions will not be far off.

## Conclusions

The clinical outlook remains favorable for beryllium-sensitized individuals over the first 5-12 years. However, declines in D_L_CO may presage further and more serious clinical manifestations in the future. These conclusions are tempered by the possibility of selection bias and other study limitations.

## Competing interests

We received funding from Brush-Wellman Inc., Cleveland, Ohio to conduct this study. Dr. Deubner is a full time employee of at this company. As part of the initial agreement with the company the findings were not disclosed or discussed with them till all the data was collected and analysis was done. Hence influence of the funding on our findings is minimal if any.

Stocks/Shares: Dr. Duebner has stock and stock options in the company, and may receive bonus salary tied to company economic performance, rest of the authors do not hold any stocks or shares in the company.

We currently do not hold any patents related this article or plan to apply for the same in the future.

## Authors' contributions

MC designed the study and wrote the paper, MD collected, analyzed and interpreted the data. All authors reviewed the paper and discussed the results and implications and commented on the manuscript at all stages.

## Pre-publication history

The pre-publication history for this paper can be accessed here:

http://www.biomedcentral.com/1471-2458/10/5/prepub
